# Supplementary material for: Allosteric substrate release by a sialic acid TRAP transporter substrate binding protein
Source: Commun Biol. 2024 Nov 23;7:1559. doi: 10.1038/s42003-024-07263-6 (PMC11585616; doi:10.1038/s42003-024-07263-6)
Supplement: Supplementary file 1 — Supplementary Information [file 42003_2024_7263_MOESM1_ESM.pdf]

Supplementary Information for:

Allosteric substrate release by a sialic acid TRAP transporter substrate binding protein

Niels Schneberger[1], Philipp Hendricks[1], Martin F. Peter[1,2], Erik Gehrke[1], Sophie C. Binder[1], Paul-Albert Koenig[3], Stephan Menzel[3], Gavin H. Thomas[4], Gregor Hagelueken[1,*]

[1] Institute of Structural Biology, University of Bonn, Venusberg-Campus 1, 53127 Bonn, Germany

[2] Present address: Biochemistry Center, Heidelberg University, Im Neuenheimer Feld 328, 69120 Heidelberg, Germany.

[3] Core Facility Nanobodies, University of Bonn, Venusberg-Campus 1, 53127 Bonn, Germany

[4] Department of Biology (Area 10), University of York, York YO10 5YW, UK

[*]Corresponding author

E-mail: hagelueken@uni-bonn.de

Supplementary Table 1: Calculated and experimental molecular weights of SiaP-VHH complexes determined by SEC-MALS analysis.

	MWexp	MWcalc
**VcSiaP**	32.3 kDa	34.057 kDa
**NbS001**	15.2 kDa	15.776 kDa
**NbS002**	15.2 kDa	15.403 kDa
**VcSiaP-NbS001**	47.0 kDa	49.833 kDa
**VcSiaP-NbS002**	46.5 kDa	49.460 kDa
**VcSiaP-NbS001-NbS002**	61.6 kDa	65.236 kDa
**HiSiaP**	33.4 kDa	34.160 kDa
**HiSiaP-NbS003**	37.5 kDa	48.551 kDa
**HiSiaP-NbS004**	50.0 kDa	50.684 kDa
**HiSiaP-NbS005**	47.5 kDa	48.553 kDa
**HiSiaP-NbS006**	47.5 kDa	49.382 kDa
**HiSiaP-NbS009**	48.0 kDa	49.435 kDa
**HiSiaP-NbS010**	47.5 kDa	49.502 kDa
**HiSiaP-NbS011**	48.0 kDa	49.557 kDa

Supplementary Table 2: Overview on the performed ITC experiments and their results. Dissociation constants and enthalpies of binding from the presented ITC experiments. Measurements denoted by a ‘ [1)] ‘ have been performed once (n=1), all other titrations were done in technical duplicates at least (n≥2) and mean values are given including the standard deviation as error value. The binding parameters denoted by a [2)] were derived by applying a competitive model and using the given values for Neu5Ac vs. SiaP as parameters for the weak interaction [29].

	SiaP	SiaP[Neu5Ac] + VHH_x	SiaP[VHH_x] + Neu5Ac
**VHH_{VcP}#1**	K_D = 162 ± 24.8 nM<br>ΔH = -0.46 ± 0.07 kcal/mol	No binding [1)]	K_D = 209 nM [1)]<br>ΔH = -8.9 kcal/mol
**VHH_{VcP}#2**	K_D = 13.0 ± 2.31 nM<br>ΔH = -9.1 ± 0.16 kcal/mol	K_D = 1.97 ± 0.31 nM [2)]<br>ΔH = -9.0 ± 0.09 kcal/mol	No binding
**VHH_{VcP}#4**	K_D = 2.66 nM [1)]<br>ΔH = -9.34 kcal/mol	K_D = 1.91 nM [1)]<br>ΔH = -9.37 kcal/mol	K_D = 46.1 nM [1)]<br>ΔH = -17.6 kcal/mol
**VHH_{VcP}#5**	K_D < 1 nM [1)]<br>ΔH = -14.5 kcal/mol	K_D = 4.73 nM [1)]<br>ΔH = -14.7 kcal/mol	K_D = 53.9 nM [1)]<br>ΔH = -18.3 kcal/mol
**VHH_{VcP}#6**	K_D < 1 nM [1)]<br>ΔH = -21.8 kcal/mol	K_D = 6.25 nM [1)]<br>ΔH = -21.8 kcal/mol	K_D = 56.8 nM [1)]<br>ΔH = -17.6 kcal/mol
**VHH_{VcP}#9**	K_D = 3.54 nM [1)]<br>ΔH = -14.5 kcal/mol	K_D = 16.1 nM [1)]<br>ΔH = -13.2 kcal/mol	K_D = 364 nM [1)]<br>ΔH = -16.5 kcal/mol
**VHH_{VcP}#10**	K_D = 7.49 nM [1)]<br>ΔH = -10.9 kcal/mol	K_D = 51.7 nM [1)]<br>ΔH = -12.2 kcal/mol	K_D = 59.9 nM [1)]<br>ΔH = -17.3 kcal/mol
**VHH_{VcP}#11**	K_D = 12.3 nM [1)]<br>ΔH = -14.4 kcal/mol	K_D = 1.59 nM [1), 2)]<br>ΔH = -12.5 kcal/mol	No binding [1)]

**No binding observed**	Neu5Ac vs. VcSiaP:	K_D = 211 ± 38.9 nM<br>ΔH = -10.8 ± 0.3 kcal/mol
**Decreased affinity**	Neu5Ac vs. HiSiaP:	K_D = 48.2 ± 6.98 nM<br>ΔH = -18.7 ± 1.4 kcal/mol
**Competitive effect**		

	$VcSiaP_{W73A}$ (W73A)	W73A [Neu5Ac] + VHH_x	W73A [VHH_x] + Neu5Ac
**VHH_{VcP}#2**	K_D = 22.8 ± 14.0 nM<br>ΔH = -5.5 ± 0.04 kcal/mol	K_D = 5.41 ± 5.53 nM<br>ΔH = -8.5 ± 0.3 kcal/mol	K_D = 1200 nM [1)]<br>ΔH = -13.4 kcal/mol
		Neu5Ac vs. $VcSiaP_{W73A}$:	K_D = 76.9 ± 10.5 nM<br>ΔH = -12.5 ± 0.1 kcal/mol

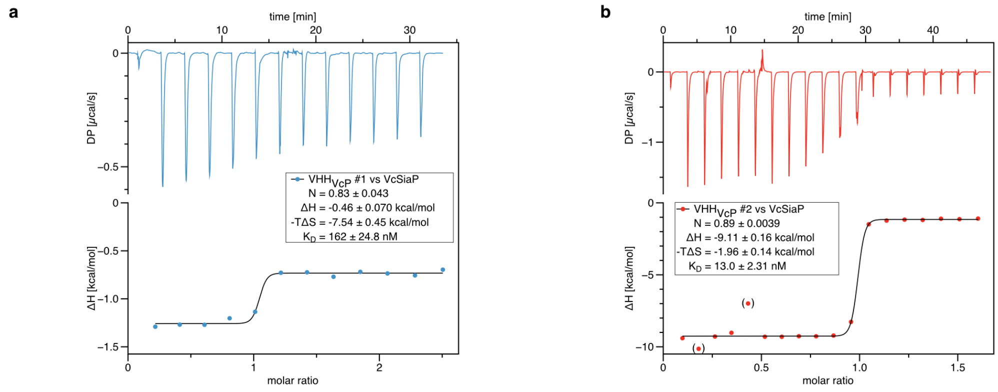

Supplementary Fig. 1: ITC measurements for VcSiaP and its specific VHHs. a) Thermogram of the titration of VHH_VcP#1 to VcSiaP (top) and the resulting binding curve (bottom). For the thermogram the differential power (DP) is plotted against the experiment time and for the binding curve, the binding enthalpy (ΔH) is shown on the y-axis and the molar ratio on the x-axis. The averaged binding parameters from three independent measurements (n=3, technical replicates) are listed. **b)** The same as in a) but for the titration of VHH_VcP#2 to VcSiaP. The data points in parentheses have not been used for calculating the fit. The titration of VHH_VcP#2 vs VcSiaP was performed four times (n=4, technical replicates).

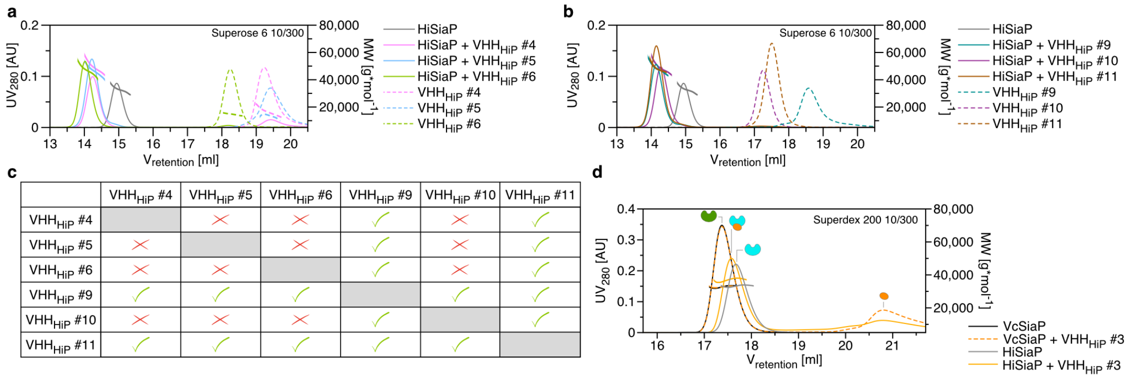

	VHH_{HiP} #4	VHH_{HiP} #5	VHH_{HiP} #6	VHH_{HiP} #9	VHH_{HiP} #10	VHH_{HiP} #11
VHH_{HiP} #4		✗	✗	✓	✗	✓
VHH_{HiP} #5	✗		✗	✓	✗	✓
VHH_{HiP} #6	✗	✗		✓	✗	✓
VHH_{HiP} #9	✓	✓	✓		✓	✓
VHH_{HiP} #10	✗	✗	✗	✓		✓
VHH_{HiP} #11	✓	✓	✓	✓	✓	

Supplementary Fig. 2: SEC MALS measurements for HiSiaP and its specific VHHs and epitope binning. a) SEC MALS measurements for the individual heterodimeric HiSiaP–VHH complexes including VHH_HiP#4 (magenta), VHH_HiP#5 (blue), and VHH_HiP#6 (green). Runs of all individual components are included as reference (VHHs as dashed lines, HiSiaP as solid grey line). **b)** Same as in a) but for VHH_HiP#9 (teal), VHH_HiP#10 (purple), and VHH_HiP#11 (brown). For the individual runs with only one of the VHH, only the curves for UV280 absorption are shown. **c)** Overview of all possible 1:1:1 complexes of two individual VHHs and HiSiaP. Showing three individual binding epitopes, an overlapping one for VHH_HiP#4, VHH_HiP#5, VHH_HiP#6, VHH_HiP#10, and an individual one for VHH_HiP#9 and VHH_HiP#11, respectively. **d)** Same as in a) and b) but for VHH_HiP#3 + VcSiaP (yellow dashed line) and VHH_HiP#3 + HiSiaP (yellow line), respectively. VcSiaP (black) and HiSiaP (grey) without VHH were run as control. Pictograms above the graph represent the corresponding proteins and complexes. All experiments were done once (n=1).

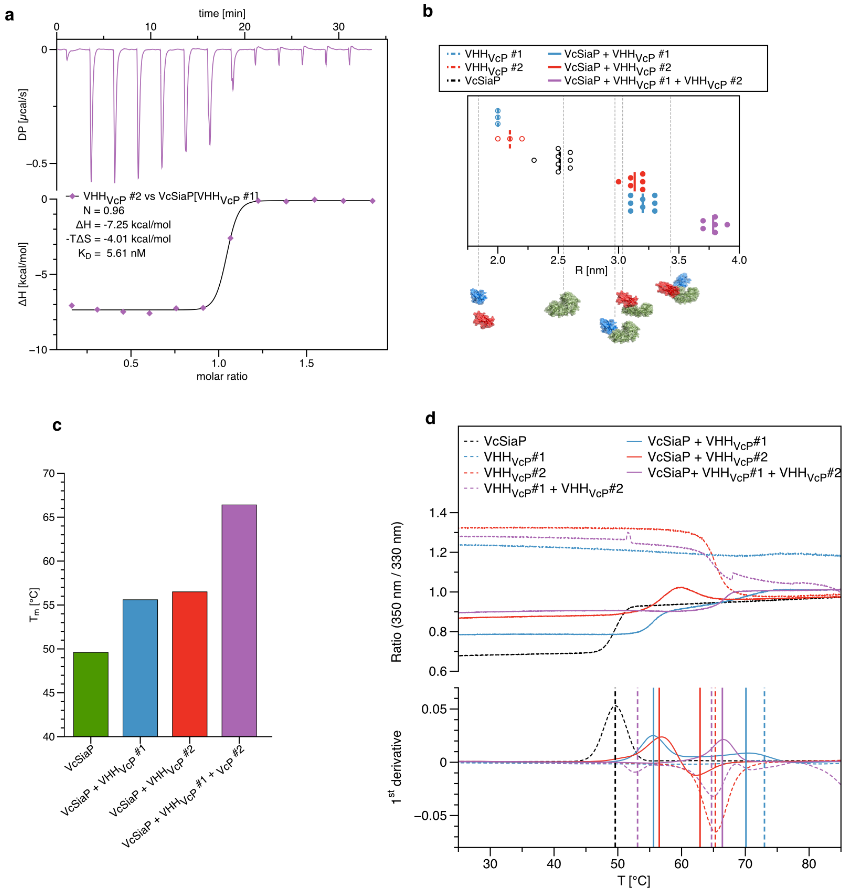

Supplementary Fig. 3: Additional information on heterodimeric and heterotrimeric VcSiaP–VHH complexes. a) Thermogram of the titration of VHH_{VcP} #2 to VcSiaP[VHH_{VcP} 1] (top) and the resulting binding curve (bottom). For the thermogram the differential power (DP) is plotted against the experiment time and for the binding curve, the binding enthalpy (ΔH) is shown on the y-axis and the molar ratio on the x-axis. The resulting binding parameters are listed. This experiment was done once (n=1). **b)** Hydrodynamic radii of all possible heterodimeric and heterotrimeric VcSiaP–VHH complexes and all individual components. Each dot corresponds to one individual measurement which includes 20 single acquisitions á 3 seconds. The calculated radii from the crystal structures are represented by dashed grey lines and the corresponding pictogram. All experiments were done in technical replicates. VHHs only (n=1), VcSiaP only (n=3), VcSiaP + VHHVcP#1 (n=3), VcSiaP + VHHVcP#2 (n=2), VcSiaP + VHHVcP#1 + VHHVcP#2 (n=2). Thermal nanoDSF measurements revealed that both 1:1 VcSiaP–VHH complexes are thermally stabilized by about 6 °C compared to VcSiaP. For the heterotrimeric complex, a further increased denaturation temperature was observed. **d)** Thermograms of different proteins and their complexes showing the intensity ratio of 350nm fluorescence to 330nm fluorescence (upper half) and the corresponding 1st derivatives of these curves (lower half). All curves are color-coded as explained in the caption on top. The vertical lines indicate the melting temperatures shown in c). The nanoDSF experiment was done as technical duplicate (n=2).

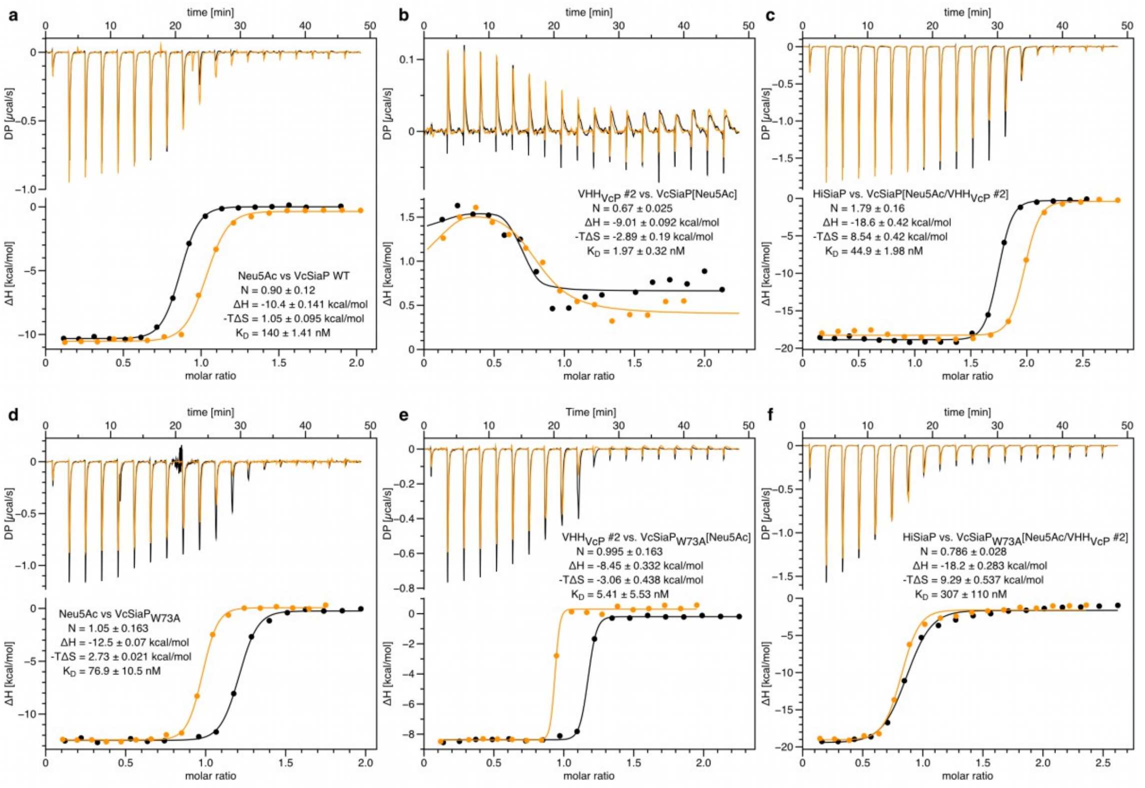

Supplementary Fig. 4: ITC measurements for VcSiaP and its specific VHHs. a) - c) Both replicates of the measurement presented in Fig. 3 b)-d). **d) - f)** The same experiments as shown in a) - c), but using the VcSiaP W73A mutant instead of the wild-type protein. Therefore, no competitive effect was observed for the titration of VHH_Vc#2 to VcSiaP W73A[Neu5Ac] (e). One measurement series is shown in orange and one in black (n=2, technical replicate).

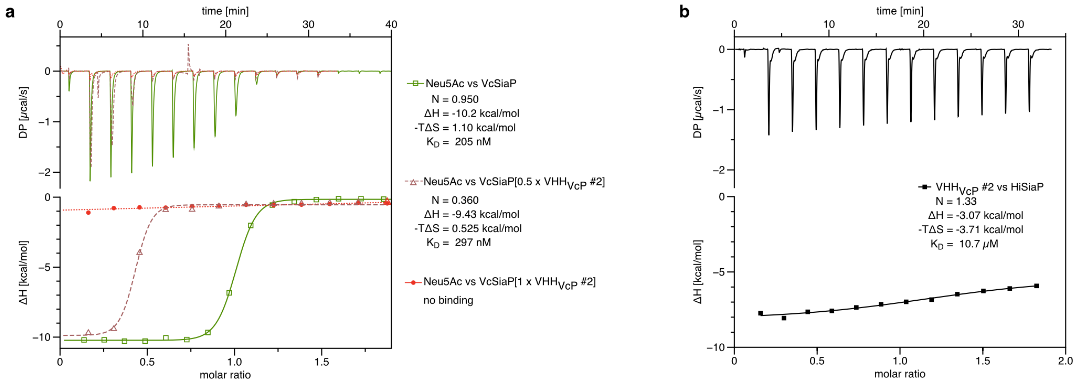

Supplementary Fig. 5: Inhibitory effect of VHH_VcP#2 on VcSiaP substrate binding. a) ITC experiments showing that VcSiaP loaded with an equimolar amount of VHH_VcP#2 cannot bind Neu5Ac (red). The same experiment, but preincubating VcSiaP with a sub-molar amount of VHH_VcP#2, shows that P-domain which is not in complex with the VHH is able to bind Neu5Ac (dashed red line). The titration of Neu5Ac towards VcSiaP (green) is included as a reference. The binding parameters calculated from the corresponding curves are shown on the right. The titration of Neu5Ac to VcSiaP was performed n>5, biological replicates, the titration of Neu5Ac to VcSiaP + VHH_{VcP} #2 (1:1) was done twice (n=2, technical replicates) and the titration of Neu5Ac to VcSiaP + VHH_{VcP} #2 (1:0.5) was done once (n=1). **b)** ITC experiment titrating VHH_VcP_#2 to HiSiaP revealed no obvious interaction. However, the calculated binding affinity is in the micromolar range and thus a factor of 1,000 weaker than the VcSiaP binding. The experiment was done twice (n=2, technical replicates).

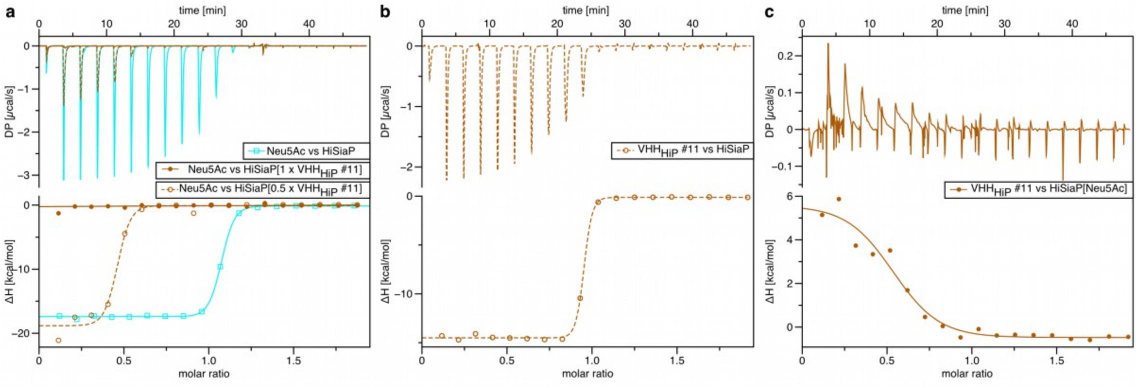

Supplementary Fig. 6: Inhibitory effect of VHH_HiP#11 on HiSiaP substrate binding. a) ITC experiments showing that HiSiaP loaded VHH_HiP#11 inhibits Neu5Ac binding. An equimolar amount of VHH (solid brown) or a sub-molar amount of VHH (dashed brow) was preincubated with the P-domain. The titration of Neu5Ac towards HiSiaP (cyan) is included as a reference. **b)** ITC data for the binding of VHH_{HiP} #11 to HiSiaP in the absence of sialic acid. **c)** ITC data for the binding of VHH_{HiP} #11 to substrate bound HiSiaP. All experiments were done once (n=1).

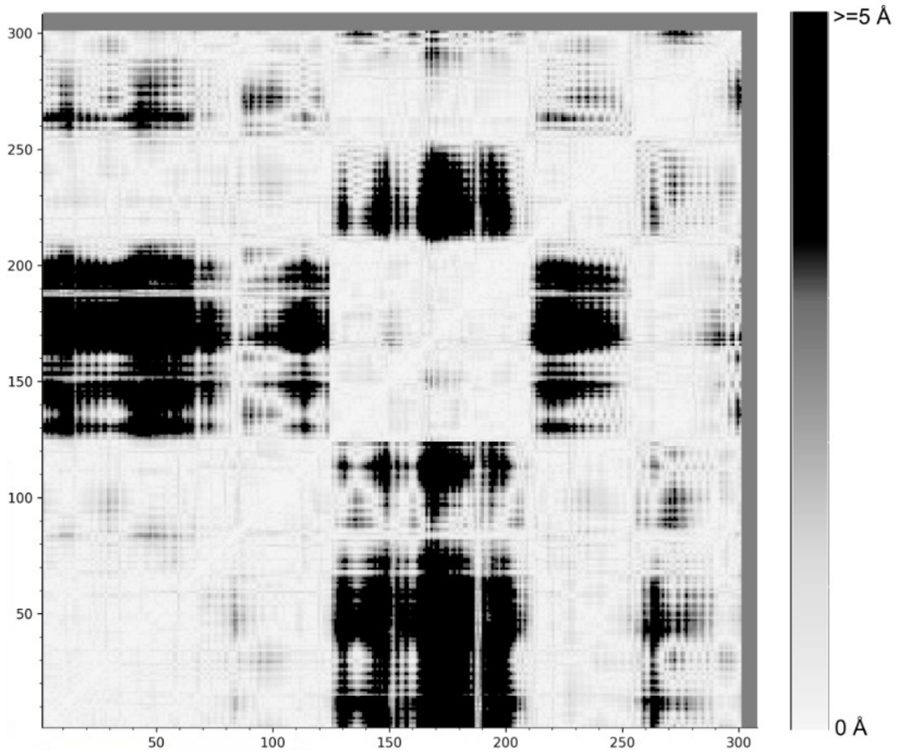

Supplementary Fig. 7: Difference distance matrix of VcSiaP in its open- and closed-state. The white to black gradient visualizes the positional change between each pair of residues of VcSiaP. A magnification of this matrix that focusses on the N-terminal lobe of the P-domain is included in Fig. 6 a.

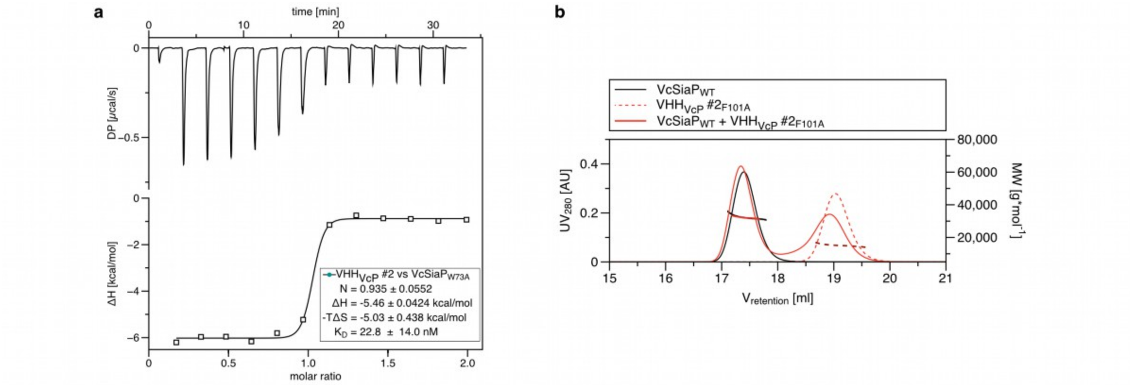

Supplementary Fig. 8: Binding behavior of VHH_VcP#2 to VcSiaP W73A and VHH_VcP#2 F101A to VcSiaP. a) ITC experiments showing that VHH_VcP#2 binds to the W73A mutant of VcSiaP in the same order of magnitude as to the wild-type. The experiment was done twice (n=2, technical replicates). **b)** SEC MALS analysis revealed that an F101A mutant of VHH_VcP#2 binds VcSiaP weakly. The SEC MALS measurement was done once (n=1).

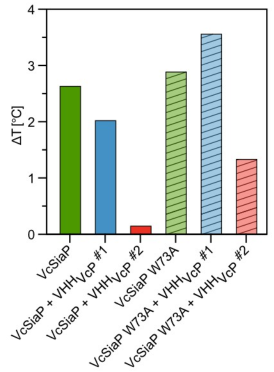

Supplementary Fig. 9: Thermal stabilization of sialic acid on VcSiaP and its VHH-complexes. While wild type VcSiaP bound to VHH_VcP#2 cannot be stabilized by addition of sialic acid, this behavior can be recovered by mutating W73 to an alanine. All measurements were performed as technical duplicates (n=2).

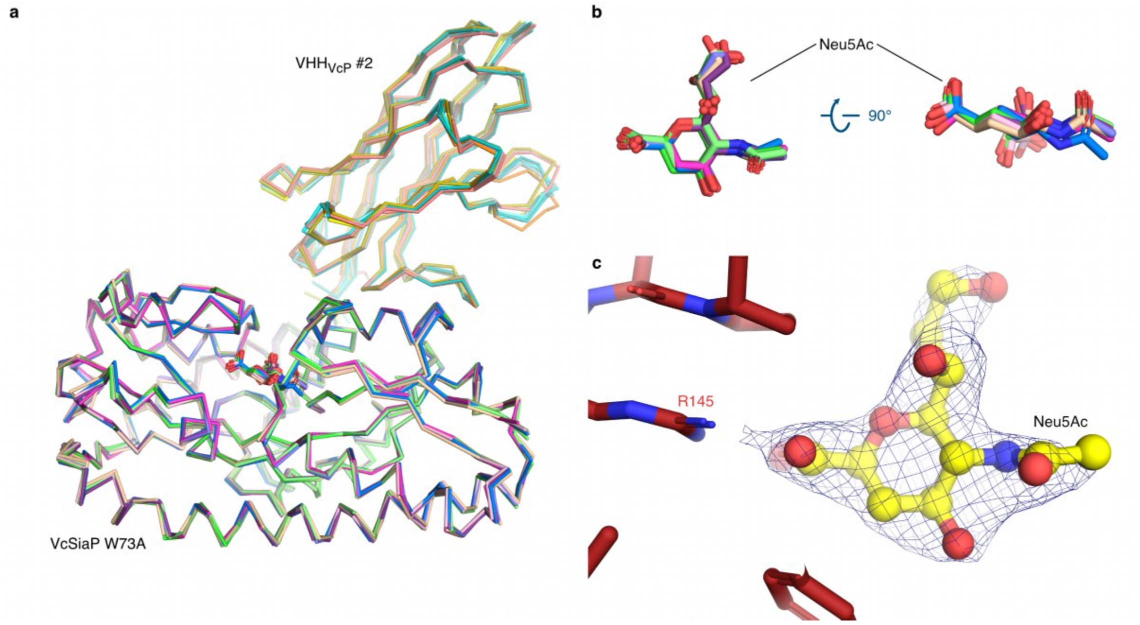

Supplementary Fig. 10: Crystal Structure of VcSiaP W73A bound to Neu5Ac and VHH_VcP#2. a) Superposition of all 12 individual VcSiaP–VHH_VcP#2 complexes within the asymmetric unit. Each dimer was handled as one object and superimposed on the basis of the P-domain. The protein chains are shown as ribbon representation and sialic acid in the binding pocket as sticks. **b)** Magnification of the bound sialic acid from the superposition shown in a). **c)** Neu5A (yellow ball and stick model) is shown inside its electron density and the conserved interaction between Arg145 and the carboxylic acid group can be seen.

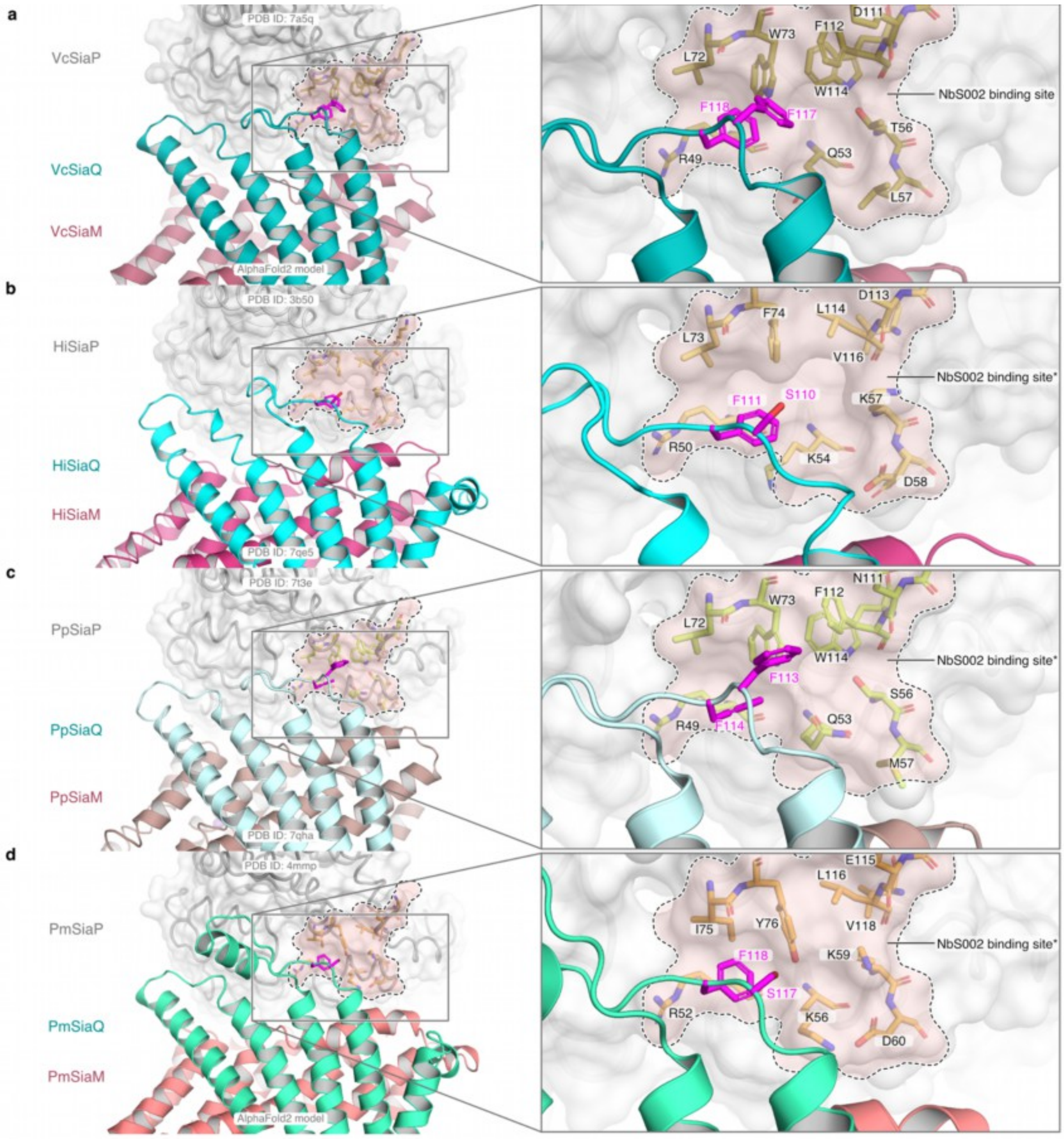

Supplementary Fig. 11: The hydrophobic surface area that is part of the VHH_{VcP} #2 binding site, is conserved among sialic acid TRAP transporters.

a-d) Model of the tripartite SiaPQM complex from different organisms as shown in Fig. 8 for VcSiaPQM (a) and HiSiaPQM (b). For reasons of clarity, the TRAP transporters from *Vibrio cholerae* (a) and *Haemophilus influencae* (b) are again included. The tripartite complexes from *Photobacterium profundum* (c) and *Pasteurella multocida* (d) are depicted in the same way. All models were built up from AlphaFold2 predictions and experimental structures as annotated. The binding area of VHH_{VcP} #2 is highlighted and labelled. The Q (teal) and M(red) transmembrane domains are depicted as cartoon model and the P-domain is shown as surface representation. On the right-hand-side, a magnification of the region of interest is shown and the highlighted amino acid residues are labeled.
