# Supplementary material for: Allosteric substrate release by a sialic acid TRAP transporter substrate binding protein
Source: Commun Biol. 2024 Nov 23;7:1559. doi: 10.1038/s42003-024-07263-6 (PMC11585616; doi:10.1038/s42003-024-07263-6)
Supplement: Supplementary file 2 — Description of Additional Supplementary Materials [file 42003_2024_7263_MOESM2_ESM.pdf]

Description of Additional Supplementary Files

File name: Supplementary Data 1

Description: source data for all graphs
