# Supplementary material for: Allosteric substrate release by a sialic acid TRAP transporter substrate binding protein
Source: Commun Biol. 2024 Nov 23;7:1559. doi: 10.1038/s42003-024-07263-6 (PMC11585616; doi:10.1038/s42003-024-07263-6)
Supplement: Supplementary file 4 — Reporting Summary [file 42003_2024_7263_MOESM4_ESM.pdf]

Corresponding author(s): Gregor Hagelueken

Last updated by author(s): Oct 29, 2024

Reporting Summary

Nature Portfolio wishes to improve the reproducibility of the work that we publish. This form provides structure for consistency and transparency in reporting. For further information on Nature Portfolio policies, see our Editorial Policies and the Editorial Policy Checklist.

Statistics

For all statistical analyses, confirm that the following items are present in the figure legend, table legend, main text, or Methods section.

n/a	Confirmed	
☐	☒	The exact sample size (*n*) for each experimental group/condition, given as a discrete number and unit of measurement
☐	☒	A statement on whether measurements were taken from distinct samples or whether the same sample was measured repeatedly
☒	☐	The statistical test(s) used AND whether they are one- or two-sided<br>*Only common tests should be described solely by name; describe more complex techniques in the Methods section.*
☒	☐	A description of all covariates tested
☒	☐	A description of any assumptions or corrections, such as tests of normality and adjustment for multiple comparisons
☐	☐	A full description of the statistical parameters including central tendency (e.g. means) or other basic estimates (e.g. regression coefficient) AND variation (e.g. standard deviation) or associated estimates of uncertainty (e.g. confidence intervals)
☒	☐	For null hypothesis testing, the test statistic (e.g. *F*, *t*, *r*) with confidence intervals, effect sizes, degrees of freedom and *P* value noted<br>*Give P values as exact values whenever suitable.*
☒	☐	For Bayesian analysis, information on the choice of priors and Markov chain Monte Carlo settings
☐	☐	For hierarchical and complex designs, identification of the appropriate level for tests and full reporting of outcomes
☒	☐	Estimates of effect sizes (e.g. Cohen's *d*, Pearson's *r*), indicating how they were calculated

Our web collection on statistics for biologists contains articles on many of the points above.

Software and code

Policy information about availability of computer code

Data collection	Software for diffraction data collection at beamline P13: mxCube v2
Data analysis	autoproc (v 1.1.7), Phenix (v. 1.19.2), coot (v 0.9.8.8), PyMOL (v 2.3.4), Molprobity (v. 4.5.2), XDS (Version January 31, 2020), ChimeraX (v 1.7.1), ISOLDE (v 1.6)

For manuscripts utilizing custom algorithms or software that are central to the research but not yet described in published literature, software must be made available to editors and reviewers. We strongly encourage code deposition in a community repository (e.g. GitHub). See the Nature Portfolio guidelines for submitting code & software for further information.

Data

Policy information about availability of data

All manuscripts must include a data availability statement. This statement should provide the following information, where applicable:

- Accession codes, unique identifiers, or web links for publicly available datasets
- A description of any restrictions on data availability
- For clinical datasets or third party data, please ensure that the statement adheres to our policy

The coordinate and diffraction data generated in this sutdy have been deposited in the PDB under accession codes XXXX, YYY, ZZZ (doi)s.

Research involving human participants, their data, or biological material

Policy information about studies with human participants or human data. See also policy information about sex, gender (identity/presentation), and sexual orientation and race, ethnicity and racism.

Reporting on sex and gender	Not applicable for this study.
Reporting on race, ethnicity, or other socially relevant groupings	Not applicable for this study.
Population characteristics	Not applicable for this study.
Recruitment	Not applicable for this study.
Ethics oversight	Not applicable for this study.

Note that full information on the approval of the study protocol must also be provided in the manuscript.

Field-specific reporting

Please select the one below that is the best fit for your research. If you are not sure, read the appropriate sections before making your selection.

☒ Life sciences ☐ Behavioural & social sciences ☐ Ecological, evolutionary & environmental sciences

For a reference copy of the document with all sections, see nature.com/documents/nr-reporting-summary-flat.pdf

Life sciences study design

All studies must disclose on these points even when the disclosure is negative.

Sample size	No sample size calculations were performed. No experiments involving biological specimens were examined such that sample size does not apply. Dozens of crystals were screend to identify the optimal crystal for data collection. The number of crystals screened was random and was not<br>limited by any experimental parameter. Biochemical experiments were confirmed with multiple replicates as detailed in the Methods and Figure Legends.
Data exclusions	No data was excluded.
Replication	The results of the biochemical experiments could be confirmed by multiple successfull replications as detailed in Methods and in the figure legends.
Randomization	No experiment involving animals or humans was performed in this study, therefore randomization is not applicable for this study
Blinding	Blinding is not applicable for this study as no experiment involving humans or animals was performed

Reporting for specific materials, systems and methods

We require information from authors about some types of materials, experimental systems and methods used in many studies. Here, indicate whether each material, system or method listed is relevant to your study. If you are not sure if a list item applies to your research, read the appropriate section before selecting a response.

Materials & experimental systems

n/a	Involved in the study	
☐	☒	Antibodies
☒	☐	Eukaryotic cell lines
☒	☐	Palaeontology and archaeology
☒	☐	Animals and other organisms
☒	☐	Clinical data
☒	☐	Dual use research of concern
☒	☐	Plants

Methods

n/a	Involved in the study	
☒	☐	ChIP-seq
☒	☐	Flow cytometry
☒	☐	MRI-based neuroimaging

Antibodies

Antibodies used	The VHH proteins were produced by immunization of an alpaca with the HiSiaP or VcSiaP proteins, as described in the Methods section.
Validation	The validation of the VHHs is one of the central points of this manuscript.

Plants

Seed stocks	Not applicable for this study.
Novel plant genotypes	Not applicable for this study.
Authentication	Not applicable for this study.
